# Herbal Extract Mixture Modulates Intestinal Antioxidative Capacity and Microbiota in Weaning Piglets

**DOI:** 10.3389/fmicb.2021.706758

**Published:** 2021-07-28

**Authors:** Meiwei Wang, Huijun Huang, Lei Wang, Huansheng Yang, Shengwen He, Feng Liu, Qiang Tu, Shanping He

**Affiliations:** ^1^State Key Laboratory of Developmental Biology of Freshwater Fish, Hunan Provincial Key Laboratory of Animal Intestinal Function and Regulation, Hunan Normal University, Changsha, China; ^2^Anhui Tianan Biotechnology Company Limited, Luan, China; ^3^Yucheng Baolikang Biological Feed Company Limited, Dezhou, China

**Keywords:** colonic microbiota, herbal extract mixture, intestinal antioxidant capacity, weaning piglets, Nrf2 pathway

## Abstract

Recently, herbal extracts have been applied in multiple aspects, such as medicine and animal feed. Different compositions of herbal extract mixture (HEM) have various components and diverse functions. This study aimed to evaluate the effects of HEM (*Lonicera japonica, Astragalus membranaceus*, *Eucommia folium*, and *Codonopsis pilosula*) on intestinal antioxidant capacity and colonic microbiota in weaned pigs. A total of 18 piglets [Duroc × (Landrace × Yorkshire)] with the initial body weight of 5.99 ± 0.13 kg (weaned at 21 days) were randomly divided into two groups (*n* = 9): the control group (CON, basal diet) and the HEM treatment group (HEM, 1,000 mg/kg HEM + basal diet). The experiment period lasted for 14 days. Our results showed that dietary supplementation with HEM modulated the antioxidant capacity through decreasing the activity of superoxide dismutase (SOD) in the ileum and glutathione peroxidase (GSH-PX) in the serum, and decreasing the mRNA expression of Kelch like-ECH-associated protein 1 (Keap1) in the jejunum and the protein level of Keap1 in the ileum. Moreover, the HEM group modified the composition of colonic microbiota with affecting relative abundances of the Firmicutes and Bacteroidetes at the phylum level. Taken together, supplementation of HEM can regulate the antioxidant capacity and modify the composition of colonic bacteria in weaning piglets. This study provides new insights into the combination effects of herbal extracts on weaning piglets.

## Introduction

Early weaning is a beneficial practice for improving sow reproductive performance in pig industry (Robert et al., 1999). However, early weaning reduces the growth performance of weaning piglets. Moreover, early weaning damages the intestinal function and gives rise to poor immunity status, thereby leading to weaning stress on early weaning piglets (Blecha et al., 1983). Disruption of intestinal function after weaning is correlated with shorter intestinal villus, hyperplasia of the intestinal crypt, disrupted intestinal barrier, and decreased digestive capacity (Smith et al., 2010; Yang et al., 2013). Moreover, because of the insufficient immune function of weaning piglets, they are easy to suffer weaning stress incurred by pathogens in feed (McLamb et al., 2013). Early weaning also destroys the antioxidant system and leads to excessive reactive oxygen species, thus resulting in intestinal oxidative stress in weaning piglets (Lauridsen, 2019). Weaning stress increases harmful bacteria to colonize in the intestine and disrupts intestinal homeostasis (Gresse et al., 2017). At present, functional amino acids, plant extracts, and organic acids are commonly used to alleviate weaning stress in swine farms (Jayaraman and Nyachoti, 2017; Modina et al., 2019).

In livestock production, plant extracts have been recommended as feed additives to substitute antibiotics because they are natural products and have various beneficial activities, including antiviral, antibacterial, and antioxidant activities (Liu et al., 2018). Liu et al. (2016) have reported that the dietary supplementation of *Scutellaria baicalensis* and *Lonicera japonica* extract mixture (0.025 and 0.05%) for 12 weeks improves the growth performance through increasing the overall average daily growth (ADG) and the ratio of gain to feed, elevates the nutrient digestibility of nitrogen, energy, and dry matter, decreases the serum cortisol concentration, and improves meat quality through increasing the pH of meat and decreasing the concentration of 2-thiobarbituric acid in finishing pigs. Manzanilla et al. (2004) reported that plant extract mixture (containing 5% *carvacrol* extracted from oregano, 3% *cinnamaldehyde* extracted from cinnamon, and 2% *capsicum oleoresin* extracted from Mexican pepper) and formic acid in the diet modify the gastrointestinal ecosystem through increasing the ratio of lactobacilli to enterobacteria, increase stomach contents, and decrease the stomach emptying rate through extending the gastric retention time of weaning piglets. Dietary supplementation of herbal extract (0.75% inclusion, including *cinnamon*, *thyme*, and *oregano* extract) affects the composition of gut microbiota through inhibiting the proliferation of coliform bacteria and increases the pH of colon in weaning piglets (Namkung et al., 2004). These studies suggested that herbal extract mixtures have different effects on the physiology of piglets.

Golden-and-silver honeysuckle (*Lonicera japonica*), huangqi (*Astragalus membranaceus*), duzhong leaves (*Eucommia folium*), and dangshen (*Codonopsis pilosula*) contain various active ingredients, including organic acids, isoflavonoids, flavones, iridoids, polysaccharides, and sand saponins. In addition, the active ingredients of the four plant extracts have antioxidant and anti-inflammatory activities (Yen and Hsieh, 1998; He et al., 2015). However, different combinations of herbal extract mixture have different functions to weaning pigs, and the combination of several herbal extracts has more biological activity than each of the herbal extract alone. The combination of these plant extracts (*Lonicera japonica, Astragalus membranaceus*, *Eucommia folium*, and *Codonopsis pilosula*) has not been fully investigated in weaning piglets. Moreover, our previous study has shown that dietary supplementation of the herbal extract mixture (HEM), which contains golden-and-silver honeysuckle (*Lonicera japonica*), huangqi (*Astragalus membranaceus*), duzhong leaves (*Eucommia folium*), and dangshen (*Codonopsis pilosula*), improves intestinal morphology through increasing the ratio of villus height to crypt depth in the duodenum, and elevates the mRNA expression of nutrient transporters in the ileum of weaning piglets (Wang et al., 2020). Thus, in the present study, we aimed to further investigate the effects of HEM on the antioxidant capacity and colonic microbiota of weaning piglets.

## Materials and Methods

### Animals and Treatment

A total of 18 weaning piglets (Duroc × [Landrace × Yorkshire], weaned at 21 days, initial body weight = 5.99 ± 0.13 kg) were randomly allotted to two treatments: a control group with the basal diet and the HEM group with 1,000 mg/kg HEM directly added into the basal diet. The basal diet did not have any antibiotics. All piglets were raised in the same environment in the 14 days. All the pigs had *ad libitum* access to feed and water. The feeding environment was supplied, as described in the previous study (Chen S. et al., 2019). Briefly, piglets were raised in an indoor environment, which had the space of 0.5 × 1 m for movement and cleanly plastic slatted flooring (temperature: 25 ± 2°C, humidity: 65 ± 5%). The diet composition of this experiment (Supplementary Table 1) met the 2012 version NRC standard. HEM was provided by the Anhui Tianan Biotechnology Company Limited, in Luan City, China. HEM was composed of golden-and-silver honeysuckle (*Lonicera japonica*), huangqi (*Astragalus membranaceus*), duzhong leaves (*Eucommia folium*), and dangshen (*Codonopsis pilosula*).

### Sample Collection

At the end of the experiment, the piglets were anesthetized after fasting overnight as described previously (Zong et al., 2018). Briefly, all the pigs were anesthetized with the injection of a 4% sodium pentobarbital solution (40 mg/kg BW). Blood samples were collected from the precaval vein of the piglets and poured into sterile tubes. Serum samples were extracted of supernatant fluid after the centrifugation of blood (845 g, 10 min). The mesenterium was peeled off, and then the intestinal mucosal layer of the jejunum and the ileum was collected as described previously (Zong et al., 2018). Segments (10 cm) of the jejunum (5 m before the ileal–cecal junction) and the ileum (30 cm before the ileal–cecal junction) were rinsed three times with physiological saline, then the clear segments were sheared and scraped off by sterile microslides for collecting the intestinal mucosa layer, and the samples were packaged by a silver paper and rapidly frozen in liquid nitrogen. The colonic content was separately collected into two sterile tubes. All the samples were stored at an ultralow temperature freezer (−80°C), the serum and intestinal mucosal layer were analyzed for antioxidant enzyme activity and malondialdehyde (MDA) concentration, the sample of the intestinal mucosal layer was measured via the gene expression and protein expression, and the colonic content was used to analyze the intestinal bacterial and short-chain fatty acid (SCFA) concentrations.

### Determination of MDA Concentration and Antioxidant Activity

The intestinal mucosal layers of the jejunum, the ileum, and the serum were treated as described previously (Zong et al., 2018). The MDA (the thiobarbituric acid method) concentrations and enzymatic activities of superoxide dismutase (SOD, the hydroxylamine method), catalase (CAT, the ammonium molybdate method), and glutathione peroxidase (GSH-PX) were determined using commercially available kits according to the instructions of the manufacturer (Nanjing Jiancheng Bioengineering Institute, Nanjing, China).

### Quantitative Real-Time PCR (qPCR)

Quantitative real-time PCR was performed as described in our previous study (Chen S. et al., 2019). Total RNA was extracted using the Trizol reagent (Takara, Tokyo, Japan) and dissolved in diethyl pyrocarbonate (DEPC)-treated water (Sangon Biotech, shanghai, China). The quality of RNA was checked using agarose gel electrophoresis, and the concentration of RNA was measured with an Eppendorf Biophotometer (Eppendorf AG, Hamburg, Germany). cDNA was synthesized using a commercial reverse transcription kit (Takara, Tokyo, Japan) according to the instructions of the manufacturer. Briefly, first-strand cDNA was synthesized through incubating 1.0 μg total RNA with DNase I for 2 min at 42°C and reverse-transcribed using Oligo (dT) primers for 15 min at 37°C, 5 s at 85°C in a 20-μl reaction volume. Primers were designed on the National Center of Biotechnology Information (NCBI) online website based on the mRNA sequences of *Sus scrofa*. The sequences of primers used in this study were shown in Supplementary Table 2. SYBR Green mix in the quantitative real-time PCR was purchased from Thermo Scientific company (Waltham, United States). Each sample was determined with qPCR three times. The qPCR reaction mixture (10 μl) was composed of 0.3 μl forward primers (10 μM), 0.3 μl reverse primers (10 μM), 0.25 μl sample buffer, 5 μl SYBR Green (2 ×), 5 μl cDNA template (diluted fivefolds with RNase-free water), and 3.15 μl sterile water. The qPCR procedure included a 10-min pre-denaturation at 95°C and 40 cycles of amplification (denaturation at 95°C for 15 s and annealing and extension at 60°C for 20 s), followed with the melting curve program that was conducted at 60–99°C with a heating rate of 0.1°C/s. The melting curve and the amplification curve were checked to ensure the specificity of both primers and PCR products. The relative mRNA expression levels of target genes were calculated by the formula of 2^–Δ^ ^Δ^ ^Ct^ using β-actin as the internal control. The mRNA expression of the target gene was presented as the fold change to the CON group.

### Western Blotting

Western blotting was performed as described in our previous study (Wang et al., 2019). Tween-20, RIPA buffer, protease inhibitor cocktail, bicinchoninic acid assay, 5 × loading buffer, SDS-PAGE (sodium dodecyl sulfate polyacrylamide gel electrophoresis) gels kit, and horseradish peroxidase-linked secondary antibodies were purchased from Beyotime Biotechnology (Shanghai, China). β-Actin was used as the loading control, and the protein bands of each sample were determined by the Alpha Imager 2200 software (Alpha Innotech Corporation, CA, United States). The antibody against β-actin was obtained from Bimake (Shanghai, China), and the antibodies against nuclear factor erythroid 2-related factor 2 (Nrf2) and Kelch like-ECH-associated protein 1 (Keap1) were purchased from Proteintech (Wuhan, China).

### Measurement of SCFAs Concentrations

The SCFAs in the colon were analyzed according to a previous study (Zhou et al., 2019). One gram of colon digesta was diluted with distilled water, then vortexed and centrifuged at 12,000 g for 15 min. The supernatant was mixed with 25% metaphosphoric acid solution overnight, and then the fluid was centrifuged and filtered through a 0.22-μm membrane filter. The concentrations of SCFAs, including acetate, propionate, butyrate, isobutyrate, valerate, and isovalerate, were analyzed by gas chromatography (Agilent Technologies 7890B GC System; AGILENT) on a DB-FFAP column (30 m × 250 μm × 0.25 μm).

### Bacteria 16S rRNA Sequencing and Bioinformatics Analysis

The bacteria 16S rRNA sequencing of the colonic content was performed according to our previous study (Chen S. et al., 2019). The DNA of the colonic contents was extracted with the DNA Stool Mini Kit (Qiagen, Dusseldorf, Germany). The DNA sample from the colonic content and the amplification products were detected by 1% agarose gel electrophoresis. The V3–V4 region of the 16S rRNA gene was amplified by PCR using primers (forward primer, 5′-CCTACGGGNGGCWGCAG-3′; reverse primer, 5′-GACTACHVGGGTATCTAATCC-3′). The PCR mixture (20 μl) was composed of 1 μl DNA template, 2 μl deoxyribonucleotide triphosphate (TransGen Biotech, China), 0.4 μl forward primer (10 μM), 0.4 μl reverse primer (10 μM), 0.4 μl of fastpfu polymerase (TransGen Biotech, China), 4 μl of 5 × fastpfu buffer (TransGen Biotech, China), and 11.8 μl of sterile water. The PCR procedure includes pre-denaturation for 3 min at 95°C, 30 cycles of denaturation for 30 s at 95°C, annealing for 30 s at 55°C, and extension for 30 s at 72°C, followed by final extension for 6 min at 72°C. The PCR products were purified with a GeneJET Gel Extraction kit (Thermo Fisher Scientific, Waltham, United States). Purified DNA was subjected to paired-end sequencing on the Illumina MiSeq platform (Illumina, Sand Diego, CA, United States) according to the instructions of the manufacturer. Illumina MiSeq sequencing, processing of sequencing data, and bioinformatics analysis were performed by Novogene Bioinformatics Technology Co., Ltd. (Beijing, China). High-quality clean reads were obtained through filtering the adapters and low-quality reads (such as trailing quality score < 20, reads with primer mismatches > 2) according to the quality-controlled process. Raw tags, clean tags, effective tags, and good coverage were provided by the commercial service company. Operational taxonomic unit (OTU, sequence similarity of 97%) clustering and species taxonomy were analyzed by the Uparse software. Based on OTU clustering results, the alpha diversity of Shannon and Chao1 was analyzed. The beta diversity of principal coordinates analysis (PCOA) and principal component analysis (PCA) was generated through calculating the distance of unweighted Unifrac. Based on OTU clustering results, the information of relevant species and the species abundance on the top 10 in the phylum and genus level was obtained. The statistical data of the linear discriminant analysis effect size (LEfSe) was analyzed using the linear discriminant analysis to assess species with significant difference among treatments. The environmental factor correlation analysis was performed using the R software (psych and heatmap package) on the online platform of Novogene Bioinformatics Technology Co., Ltd. (Beijing, China).

### Statistical Analysis

All the data in the present study were expressed as means ± SEM and analyzed using the SPSS software (SPSS Inc., Chicago, IL, United States) through Student’s *t*-test. All the statistical data of the colonic bacterial community in the phylum and genus were analyzed by the Mann–Whitney *U*-test. Spearman correlations analysis was used to determine the association between environmental factors and colonic microbiota. Tables and figures in the current study were prepared by Word 2016 software (Microsoft, Redmond, United States) and GraphPad Prism 6.0 (GraphPad Software Inc., La Jolla, CA), respectively. A value of *p* < 0.05 means a statistical significance between treatments exists, while a value of *p* < 0.10 means there is a trend toward significance between treatments.

## Results

### Effect of HEM on Antioxidant Capacity

The effect of HEM on the antioxidant capacity (GSH-PX, SOD, and CAT) of weaned piglets is shown in Table 1. Compared with the CON group, the HEM group decreased the GSH-PX activity (*p* < 0.05) in the serum and the SOD activity (*p* < 0.05) in the ileum of the weaned piglets. The activity of other antioxidant enzymes and the content of MDA in both the serum and the intestine of the weaned piglets had no difference between these two groups.

**TABLE 1:**
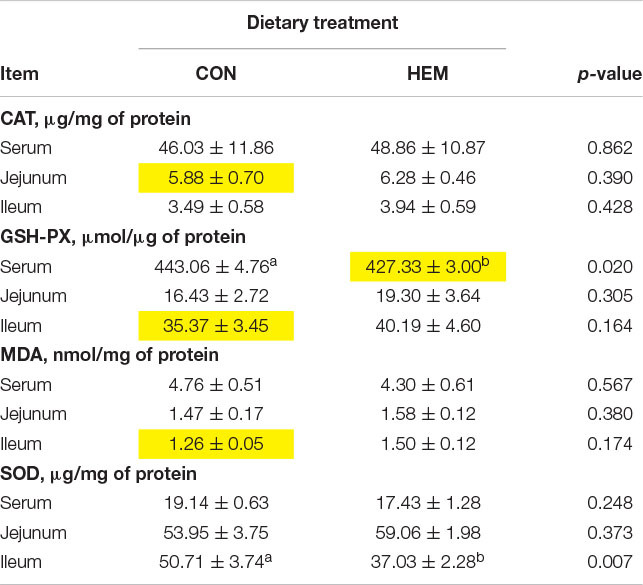
Effects of dietary supplementation with herbal extract mixture (HEM) on antioxidant indexes of weaned piglets^1^.

*^1^Values are expressed as mean ± SEM; n = 9. CON, basal diet; HEM, basal diet supplemented with 1,000 mg/kg herbal extract mixture.*

*^*a,b*^Values in the same row not sharing a common superscript differ significantly.*

### Effect of HEM on the Nrf2-Keap1 Pathway

**FIGURE 1:**
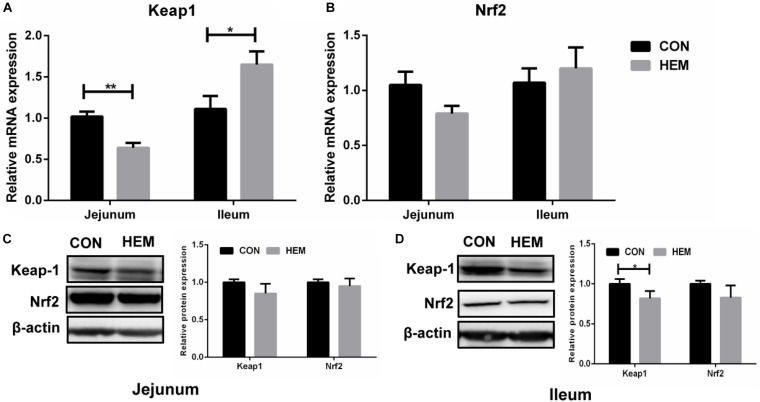
Effects of dietary supplementation with herbal extract mixture (HEM) on the Nrf2–Keap1 pathway in weaning pigs. Relative mRNA expression of Keap1 **(A)** and Nrf2 **(B)** in the jejunum and the ileum was measured by quantitative real-time PCR. Relative protein expression and quantitative analysis of Keap1 **(C)** and Nrf2 **(D)** in the jejunum and the ileum was measured by Western blotting. CON, basal diet; HEM, basal diet supplemented with 1,000 mg/kg herbal extract mixture. Data are presented as mean ± SEM, *n* = 9. *p < 0.05. 0.01 < **p < 0.05.

As shown in Figure 1, HEM significantly decreased the protein level of Keap1 (*p* < 0.05) in the ileum, while it increased the mRNA expression of Keap1 (*p* < 0.05) in the ileum of the weaned piglets compared with the CON group. In addition, HEM treatment significantly decreased the mRNA expression of Keap1 (*p* < 0.05) in the jejunum of the weaned piglets. The mRNA and protein levels of Nrf2 in both the ileum and the jejunum were similar between the CON and HEM groups.

### Effect of HEM on Colonic SCFA Concentrations

The results of colonic SCFA concentrations are shown in Table 2. The concentrations of propionate (*p* < 0.10) had a tendency to decrease in the HEM group. The concentrations of other SCFAs had no difference between the CON and HEM groups.

**TABLE 2:** Effects of dietary supplementation with herbal extract mixture (HEM) on the concentrations of short-chain fatty acid (SCFA) in the colon of weaned piglets^a^.

	Dietary treatment		
Item	CON	HEM	*p-*value
**SCFAs concentrations in colon digesta, μmol/g**			
Acetate	3.33 ± 0.13	3.06 ± 0.10	0.133
Propionate	1.72 ± 0.11	1.46 ± 0.08	0.068
Butyrate	0.88 ± 0.11	0.79 ± 0.07	0.624
Isobutyrate	0.19 ± 0.01	0.20 ± 0.01	0.516
Valerate	0.22 ± 0.03	0.20 ± 0.02	0.512
Isovalerate	0.24 ± 0.03	0.26 ± 0.02	0.687
Total SCFAs	6.58 ± 0.34	5.98 ± 0.26	0.181

*^a^Values are expressed as mean ± SEM; n = 9. CON, basal diet; HEM, basal diet supplemented with 1,000 mg/kg herbal extract mixture.*

### Effect of HEM on Colonic Microbiota

**FIGURE 2:**
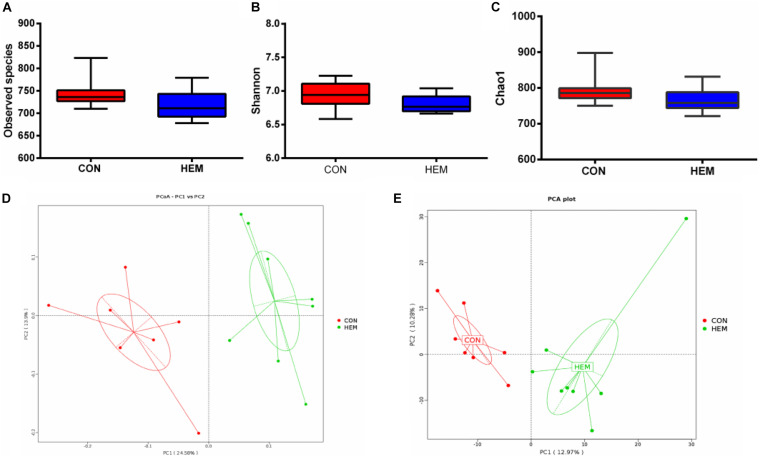
Effects of dietary supplementation with herbal extract mixture (HEM) on alpha diversity and beta diversity in weaning pigs. Alpha diversity in colonic microbiota includes observed species **(A)**, Shannon **(B)**, and Chao1 **(C)**. Beta diversity in colonic microbiota includes principal coordinates analysis **(D)** and principal component analysis **(E)**. CON (*n* = 9), basal diet; HEM (*n* = 9), basal diet supplemented with 1,000 mg/kg herbal extract mixture.

**FIGURE 3:**
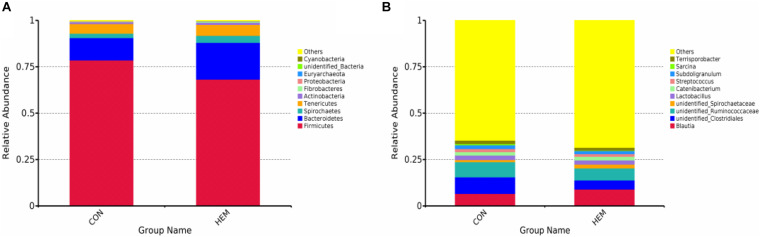
Effects of dietary supplementation with herbal extract mixture (HEM) on the colonic bacterial community structure in weaning pigs. Distribution colonic microbiota of the phylum level **(A)** and the genus level **(B)**. CON (*n* = 9), basal diet; HEM (*n* = 9), basal diet supplemented with 1,000 mg/kg herbal extract mixture.

**FIGURE 4:**
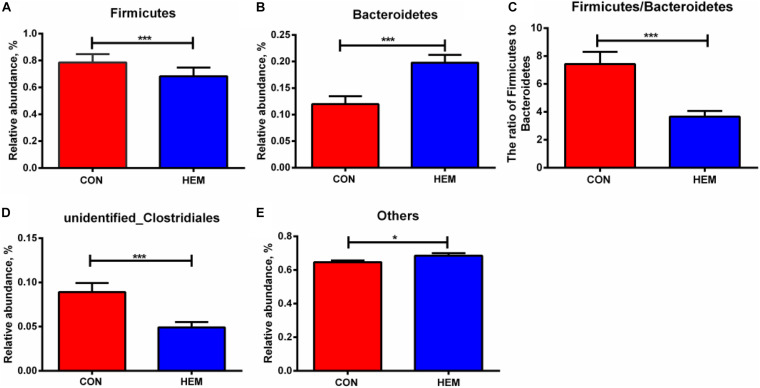
Effects of dietary supplementation with herbal extract mixture (HEM) on taxonomic differences in weaning pigs. The significantly different bacteria in the phylum of Firmicutes **(A)**, Bacteroidetes **(B)**, and the ratio of Firmicutes to Bacteroidetes **(C)** were compared between the CON and HEM groups. The significantly different bacteria in the genus of unidentified *Clostridiales* **(D)** and others **(E)** were compared between the CON and HEM groups. Data are presented as mean ± SEM. CON (*n* = 9), basal diet; HEM (*n* = 9), basal diet supplemented with 1,000 mg/kg herbal extract mixture. *p < 0.05, ***p < 0.01.

**FIGURE 5:**
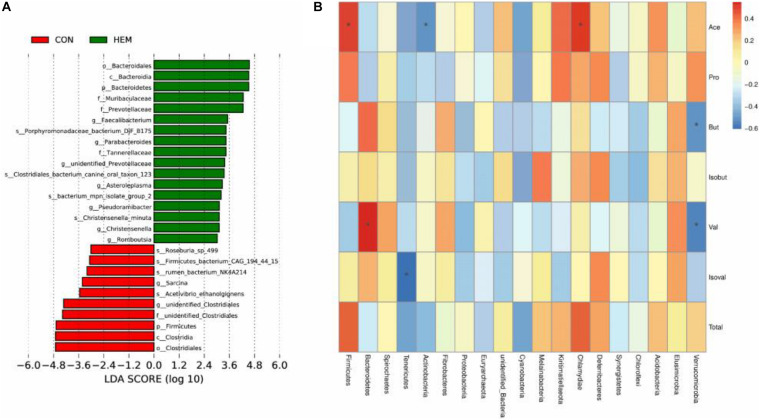
Linear discriminant analysis coupled with effect size measurements (LEfSe) analysis for the enriched microbiota **(A)** and correlations between the bacteria in the phylum and the concentrations of SCFAs in the colon **(B)**. CON (*n* = 9), basal diet; HEM (*n* = 9), basal diet supplemented with 1,000 mg/kg herbal extract mixture. *p < 0.05.

The results of colonic microbiota, including raw tags, clean tags, effective tags, and good coverage, were presented in Supplementary Table 3. These results showed that the data met the demands for further analysis. The index of observed species, the alpha diversity of Shannon, and Chao1 had the same intendency of colonic microbiota in the experiment (Figures 2A–C). In addition, the beta diversity of PCoA and PCA is shown in Figures 2D,E. These results showed that the distance between the HEM and CON groups was far because of some different species. As shown in Figure 3A, the most dominant phyla in the colon bacterium were Firmicutes, Bacteroidetes, Spirochaetes, Tenericutes, and Actinobacteria. Compared with the CON group, both the abundance of Firmicutes and the ratio of Firmicutes to Bacteroidetes were lower in the HEM groups, while the abundance of Bacteroidetes was higher in the HEM groups at the phylum level (Figures 4A–C). The distribution of the abundance at the genus level is shown in Figure 3B. The significantly different species at the genus level are shown in Figures 4D,E. Dietary supplementation with HEM significantly reduced the abundance of *unidentified Clostridiales* and increased the abundance of others species at the genus level. The bacterial biomarkers were shown with LEfSe. There were 10 specific microbes in the CON group and 17 specific microbes in the HEM group (Figure 5A). We further explored the correlation between the SCFAs and the colonic microbiota at the phylum level (Figure 5B). The abundances of Firmicutes and Chlamydiae were positively related to acetate, while the abundances of Actinobacteria were negatively related to acetate. The abundances of Verrucomicrobia were negatively associated with butyrate and valerate, while the abundances of Bacteroidetes were positively associated with valerate. In addition, the abundances of Tenericutes were negatively correlated with isovalerate.

## Discussion

Weaning stress is the main factor to cause oxidative stress in the intestine and blood of the piglets (Zhu et al., 2012). To protect against oxidative stress, the antioxidant system, including the antioxidant enzymes of SOD, CAT, and GSH-PX, reduces the production of free radicals (Bauché et al., 1994; Urso and Clarkson, 2003). The content of MDA reflects the degree of lipid peroxidation in animals (Urso and Clarkson, 2003). In this study, we found that the dietary supplementation of HEM decreased the enzyme activities of GSH-PX and SOD in the serum and the ileum, respectively. However, the content of MDA had no difference between the HEM and CON groups. Plant extracts with antioxidant capacity have been widely used in both medicine and livestock production (Jiang et al., 2014; Chen J. S. et al., 2019). For example, Jiang et al. (2014) reported that dietary supplementation with plant polyphenols (apples, grape seeds, green teas, and olive leaves) has the tendency to enhance the activities of plasma GSH-PX (day 25) and T-AOC (day 27) in the weaning piglets challenged with *Escherichia coli*. A study showed that dietary supplementation with *Lycium barbarum* polysaccharides increases the activities of SOD, CAT, and GSH-PX and decreases the content of MDA in the serum and liver of weaning piglets (Chen J. S. et al., 2019). Furthermore, dietary supplementation with *Astragalus membranaceus* root powder and *Astragalus* polysaccharides increases the total antioxidant capacity of lamb plasma but regulates the antioxidant capacity of broilers in an age-dependent manner (Zhong et al., 2012; Zhang et al., 2013). However, our results are different from previous studies. We speculate that dietary HEM alleviates weaning stress in piglets; therefore, it is not necessary for the antioxidant system to produce more antioxidant enzymes to alleviate oxidative stress. In addition, one of the most significant body defense mechanisms against oxidative stress is mediated by the Nrf2-Keap1 pathway, which stimulates the expression of genes encoding antioxidant proteins (Kobayashi and Yamamoto, 2005). Thus, we examined whether the Nrf2-Keap1 pathway is involved in mitigating oxidative stress in weaning piglets. Nrf2 is bound by the negative regulator Keap1 in the normal condition. When cells are attacked by environmental factors, such as oxidative stress, electrophiles, and chemopreventive agents, Keap1 is degraded by the ubiquitin–proteasome system, and then Nrf2 is released from Keap1, thereby stimulating the expression of antioxidant enzymes to maintain redox homeostasis (Zhang, 2006). Previous studies have shown that the plant extract containing *Magnolia officinalis*, bark extract, and *Astragalus membranaceus* activate the Nrf2 pathway to protect against oxidative stress in cells (Rajgopal et al., 2016; Adesso et al., 2018). We found that dietary HEM regulated the Nrf2–Keap1 pathway through decreasing the mRNA expression of Keap1 in the jejunum and the protein level of Keap1 in the ileum. However, our results showed that dietary HEM decreased the mRNA expression of Keap1 in the jejunum and the protein expression of Keap1 in the ileum, but increased the mRNA expression of Keap1 in the ileum. We speculated that this situation (inconsistent mRNA and protein expression of Keap1 in the ileum) might be associated with transcription and translation process, in which mRNA levels do not precisely predict protein levels in eukaryotic cells (Rojas-Duran and Gilbert, 2012).

Active compounds in the diet show a tight connection to gut microbial composition, which further influence host health (Yin et al., 2018). Previous studies have shown that dietary supplementation with herbal extracts reduces the abundances of harmful bacteria and increases the abundances of beneficial bacteria in livestock production (Namkung et al., 2004; Rahimi et al., 2011). In this study, we reported that HEM decreased the ratio of Firmicutes to Bacteroidetes through decreasing the abundance of Firmicutes and increasing the abundance of Bacteroidetes in the phylum. It is well known that gram-positive Firmicutes and gram-negative Bacteroidetes are the main bacteria in the microbial composition of host intestines (Eckburg et al., 2005). Furthermore, the ratio of Firmicutes to Bacteroidetes is associated with the overall health status (Mariat et al., 2009; Koliada et al., 2017). Koliada et al. (2017) observed that the obese people of the Ukrainian adult population have a higher ratio of Firmicutes to Bacteroidetes. Likewise, total parenteral nutrition also decreases the ratio of Firmicutes to Bacteroidetes, which is associated with intestinal Paneth cell activation in rats (Hodin et al., 2012). Our results are consistent with these findings. Our results indicated that HEM may alter the microbial composition, which could further have beneficial effects on host health. However, our results showed that HEM did not further change the bacteria of Bacteroides and Firmicutes in the genus level but changed the other bacteria in the genus level. One possible explanation for these results is that HEM supplementation modified the other bacteria that belong to Bacteroides and Firmicutes at the phylum level. A previous study indicates that intestinal SCFAs, which are produced by the gut microbiota, are involved in regulating the intestinal homeostasis (Ríos-Covián et al., 2016). Thus, we further determined the colonic concentrations of SCFAs to explore the relationship between intestinal bacteria and SCFAs. Polysaccharide from *Ganoderma atrum* increases the concentrations of SCFAs in the liver, serum, and feces of type 2 diabetic rats (Zhu et al., 2016). Dietary *Astragalus membranaceus* fiber does not modify the concentrations of SCFAs in the cecum of weaned pigs (Che et al., 2019). Similarly, our results indicated that dietary HEM did not change the concentrations of SCFAs in the colon of weaned piglets. Our results also showed that colonic bacteria had a negative or positive correlation with SCFAs.

## Conclusion

In conclusion, the dietary supplementation of the herbal extract mixture (*Lonicera japonica, Astragalus membranaceus, Eucommia folium*, and *Codonopsis pilosula*) modulates intestinal antioxidant capacity with decreasing the enzyme activity of SOD in the ileum and that of GSH-PX in the serum, and activates the Nrf2-keap1 pathway through decreasing the mRNA expression of Keap1 in the jejunum and the protein expression of Keap1 in the ileum of weaned piglets. Additionally, HEM modifies the composition of colonic microbiota with decreasing the ratio of Firmicutes to Bacteroidetes at the phylum level in weaned piglets. These findings could help to further understand the beneficial effects of HEM on the intestinal health of weaning piglets.

## Data Availability Statement

The datasets presented in this study can be found in online repositories. The names of the repository/repositories and accession number(s) can be found below: https://www.ncbi.nlm.nih.gov/, PRJNA727453.

## Ethics Statement

The animal study was reviewed and approved by the Animal Care and Use Committee of Hunan Normal University. Written informed consent was obtained from the owners for the participation of their animals in this study.

## Author Contributions

MW: methodology, investigation, data curation, writing—original draft, and funding acquisition. HH and LW: investigation and data curation. HY: validation and data curation. SWH, QT, and FL: investigation and resources. SPH: methodology, supervision, writing—review and editing, and funding acquisition. All authors contributed to the article and approved the submitted version.

## Conflict of Interest

SH was employed by the company Anhui Tianan Biotechnology Company Limited. FL and QT were employed by the company Yucheng Baolikang Biological Feed Company Limited. The remaining authors declare that the research was conducted in the absence of any commercial or financial relationships that could be construed as a potential conflict of interest.

## Publisher’s Note

All claims expressed in this article are solely those of the authors and do not necessarily represent those of their affiliated organizations, or those of the publisher, the editors and the reviewers. Any product that may be evaluated in this article, or claim that may be made by its manufacturer, is not guaranteed or endorsed by the publisher.

## Abbreviations

HEM: herbal extract mixture
CON: the control group
SOD: superoxide dismutase
GSH-PX: glutathione peroxidase
Keap1: Kelch like-ECH-associated protein 1
MDA: malondialdehyde
CAT: catalase
SCFA: short chain fatty acid
PCoA: principal co-ordinates analysis
PCA: principal component analysis
LEfSe: linear discriminant analysis coupled with effect size measurements analysis.
