# Supplementary material for: Herbal Extract Mixture Modulates Intestinal Antioxidative Capacity and Microbiota in Weaning Piglets
Source: Front Microbiol. 2021 Jul 28;12:706758. doi: 10.3389/fmicb.2021.706758 (PMC8357371; doi:10.3389/fmicb.2021.706758)
Supplement: Supplementary file 1 [file Data_Sheet_1.docx]

Supplemented table 1 Composition of experimental diets

| Component | Content(%) |
| --- | --- |
| Corn | 28.33 |
| Extruded corn | 30 |
| Soy protein concentrate | 6 |
| Soybean meal,43% CP | 8 |
| Fish meal,63% CP | 5 |
| Whey | 15 |
| Glucose | 3 |
| Soybean oil | 0.5 |
| Limestone | 0.86 |
| Calcium hydrogen phosphate | 0.4 |
| Choline chloride, 50% | 0.1 |
| Antioxidants | 0.05 |
| Citric acid | 0.8 |
| Salt | 0.1 |
| Vitamin-mineral premix | 0.45 |
| Zinc oxide | 0.2 |
| L-Lysine, 98% | 0.58 |
| DL-Methionine | 0.38 |
| L-Threonine | 0.19 |
| L-Tryptophan | 0.06 |
| Total | 100.00 |
| Calculated composition |  |
| CP | 18 |
| ME, MJ/kg | 14.2 |
| Lysine4 | 1.35 |
| Methionine4 | 0.39 |
| Cysteine4 | 0.35 |
| Threonine4 | 0.79 |
| Trptophane4 | 0.22 |

^1^HEM, diet supplemented with 1000 mg/kg herbal extract mixture.

^2^Antioxidants contained 60g/kg butylhydroquinone and 180g/kg ethoxyquinoline.

^3^Vitamin-mineral premix supplied per kilogram of feed: 10,000 IU of vitamin A, 1,000 IU of vitamin D_3_, 80 IU of vitamin E, 2.0 mg of vitamin K_3_, 0.03 mg of vitamin B_12_, 12 mg of riboflavin, 40 mg of niacin, 25 mg of d-pantothenic acid, 0.25 mg of biotin, 1.6 mg of folic acid, 3.0 mg of thiamine, 2.25 mg of pyridoxine, 300 mg of choline chloride, 150 mg of Fe (FeSO_4_), 100 mg of Zn (ZnSO_4_), 30 mg of Mn (MnSO_4_), 25 mg of Cu (CuSO_4_), 0.5 mg of I (KIO_3_), 0.3 mg of Co (CoSO_4_), 0.3 mg of Se (Na_2_SeO_3_), and 4.0 mg of ethoxyquin.

^4^Standardized ileal-digestible.

Supplemented table 2 Primers used in the experiment

| Gene | Nucleotide sequence of primers (5’–3’) | Product length (bp) |
| --- | --- | --- |
| *Keap1* | GTGTGGAGAGGAGTCTGTGTC | 112 |
|  | TCCACGTTTCTGTCTCCACG |  |
| *Nrf2* | AAGTCAGAGTCGGCTGCAT | 200 |
|  | ATTGCGCAACAGATCAACAGC |  |
| *β-actin* | AGTTGAAGGTGGTCTCGTGG | 216 |
|  | TGCGGGACATCAAGGAGAAG |  |

Supplemented table 3 Quantitative data of colonic microbiota^1^

|  | Dietary treatment | |  |
| --- | --- | --- | --- |
| Item | CON | HEM | *P-*value |
| Raw Tags | 91189.22 ± 2078.79 | 88705.11 ± 2645.37 | 0.471 |
| Clean Tags | 64011.78 ± 800.36 | 63747.67 ± 1031.80 | 0.842 |
| Effective Tags (%) | 70.47 ± 1.74 | 72.47 ± 2.78 | 0.550 |
| Good-coverage (%) | 99.86 ± 0.01 | 99.87 ± 0.01 | 0.414 |

^1^Values are expressed as mean ± SEM; n=9. CON, basal diet; HEM, basal diet supplemented with 1000 mg/kg herbal extract mixture.
